# Ten-year cumulative incidence of epiretinal membranes assessed on fundus photographs. The Beijing Eye Study 2001/2011

**DOI:** 10.1371/journal.pone.0195768

**Published:** 2018-04-26

**Authors:** Yan Yang, Yan Ni Yan, Ya Xing Wang, Jie Xu, Jie Ren, Liang Xu, Jost B. Jonas, Wen Bin Wei

**Affiliations:** 1 Beijing Tongren Eye Center, Beijing Key Laboratory of Intraocular Tumor Diagnosis and Treatment, Beijing Ophthalmology & Visual Sciences Key Lab, Beijing Tongren Hospital, Capital Medical University, Beijing, China; 2 Beijing Aier-Intech Eye Hospital, Beijing, China; 3 Beijing Institute of Ophthalmology, Beijing Ophthalmology and Visual Science Key Lab, Beijing Tongren Eye Center, Beijing Tongren Hospital, Capital Medical University, Beijing, China; 4 Department of Ophthalmology, Medical Faculty Mannheim of the Ruprecht-Karls-University Heidelberg, Mannheim, Germany; Massachusetts Eye & Ear Infirmary, Harvard Medical School, UNITED STATES

## Abstract

**Purpose:**

To assess the 10-year incidence and progression of epiretinal membranes (ERMs).

**Methods:**

The population-based longitudinal Beijing Eye Study, which included 4439 subjects (age:40+years) in 2001, was repeated in 2011 with 2695 subjects participating (66.4% of the survivors). The study participants underwent a detailed ophthalmic examination, including retinal photography. Assessing fundus photograohs, ERMs were classified as cellophane macular reflex (CMR) without retinal folds, or as preretinal macular fibrosis (PMF) without or with retinal folds.

**Results:**

Fundus photographs were available for 2476 subjects with a mean age of 69.0±7.8 years (range:51–93 years) and mean axial length of 23.3±0.9 mm (range:19.92–26.33mm). The 10-year incidence of ERMs was 8.4% (208/2476 participants; 95% confidence interval (CI):7.4,9.5). ERMs developed bilaterally in 50 (24%) individuals and unilaterally in 158 (76%) persons. The incidence of PMFs with 2.5% (95% CI: 1.9, 3.1) was lower than the incidence of CMRs with 5.9% (95% CI: 5.0, 6.9). Higher 10-year incidence of ERMs was associated with older age (*P*<0.001; odds ratio (OR): 1.06; 95%CI:1.04,1.09), previous cataract surgery (*P* = 0.003;OR:3.32;95%CI:1.51,7.29) and presence of a complete posterior vitreous detachment (*P* = 0.02;OR:1.84;95%CI:1.12,3.02). In the age groups of <60 years, 60–69 years, 70–79 years and 80+years, incidence of ERMs was 3.1%, 10.0%, 14.4% and 10.9%, respectively, with no significant gender difference.

**Conclusions:**

In Chinese aged 40+ years, the 10-year incidence of ERMs (8.4%) increased with older age, previous cataract surgery and complete posterior vitreous detachment. The ten-year incidence was lower for PMFs (2.5%) than for CMRs (5.9%).

## Introduction

Epiretinal membranes (ERM) typically occur in people aged 50 years older and are characterized by the proliferation of abnormal tissues on the inner retinal surface. They can result in mild to moderate, sometimes severe, visual impairment with an impact on quality of life [1–6]. The prevalence of idiopathic ERMs in adults has been reported to range between 1.02% to 28.9% among different ethnic groups and to increase with older age [1,7–17]. The overall prevalence of ERMs was higher in studies conducted in the United States and Australia than in investigations performed in China, Japan or Singapore [1,2,7–17]. Interestingly, the prevalence of ERMs was significantly higher in Chinese (39.0%) as compared to Hispanics (29.3%), whites (27.5%), and blacks (26.2%) in the Multi- Ethnic United States Population study, while the prevalence of ERMs in studies performed on Chinese was considerably lower in China than in the United States [11,12,14,16].

The development and progression of ERMs within a follow-up of 5 years was examined in the Blue Mountains Eye Study revealing an incidence rate of 5.3% and a progression of early ERM to an advanced stage of EMR of 9.3% [8]. Data on the incidence of ERM in Chinese have not been available so far nor did any study examine the incidence and progression of ERMs in a large population-based cohort over a follow-up of 10 years. Since ERMs are a cause for visual impairment and since the course of ERMs over a period of more than 5 years has not been examined yet in relatively large study group, we performed the present study to assess the development and progression of ERMs in a 10-year follow-up of the population-based Beijing Eye Study.

## Methods

The Beijing Eye Study is a population-based, longitudinal, cohort study performed in Greater Beijing in 2001 and repeated in 2011. According to the declaration of Helsinki, the Medical Ethics Committee of Beijing Tongren Hospital approved the study protocol and all study participants gave their written informed consent. The study was divided into an urban part and a rural part. The only eligibility criterion for the study was an age of ≥ 40 years in the year 2001. Out of 5324 eligible individuals, 4439 (83.4%) individuals participated in the baseline study in 2001. The study has been described in detail previously [18–20].

All study participants underwent an interview with standardized questions on their socioeconomic background, quality of life, psychic depression, physical activity, known major systemic diseases such as arterial hypertension and diabetes mellitus, and quality of vision. Using the mini–mental state examination or Folstein test, we assessed the cognitive function [21]. It is a 30-point questionnaire taking 5 to 10 minutes and which examines functions such as registration, attention, calculation, recall, language, ability to follow simple commands and orientation. The answers in the questionnaire are summed up to a cognitive function score (CFS) of maximal 30 points. Mild cognitive dysfunction was defined as a CFS between 23 to 19 points, moderate cognitive dysfunction as a CFS of 18 to 10 points, and severe cognitive dysfunction as a CFS ≤9 points [22]. The list of examinations performed included automatic and subjective refractometry (Auto Refractometer AR-610, Nidek Co., Ltd, Tokyo, Japan), tonometry (CT-60 computerized tonometer, Topcon Ltd., Japan), digital photography of the cornea, lens, macula and optic disc (Type CR6-45NM, Canon Inc. Tokyo, Japan), and ocular biometry applying optical low- coherence reflectometry (Lenstar 900 Optical Biometer, Haag- Streit, 3098 Koeniz, Switzerland). Fasting blood samples were examined for the concentration of blood lipids, glucose and glycosylated hemoglobin HbA1c. Blood pressure, body height and weight and the circumference of the waist and hip were meausred. For study purposes, we defined diabetes mellitus as a fasting plasma glucose concentration > = 7.0 mmol/L or by a self-reported history of physician diagnosis of diabetes mellitus or by a history of drug treatment for diabetes. Arterial hypertension was defined as systolic blood pressure ≥140 mm Hg and/or diastolic blood pressure ≥90 mm Hg, and/or self-reported current treatment for arterial hypertension.

**Fig 1:**
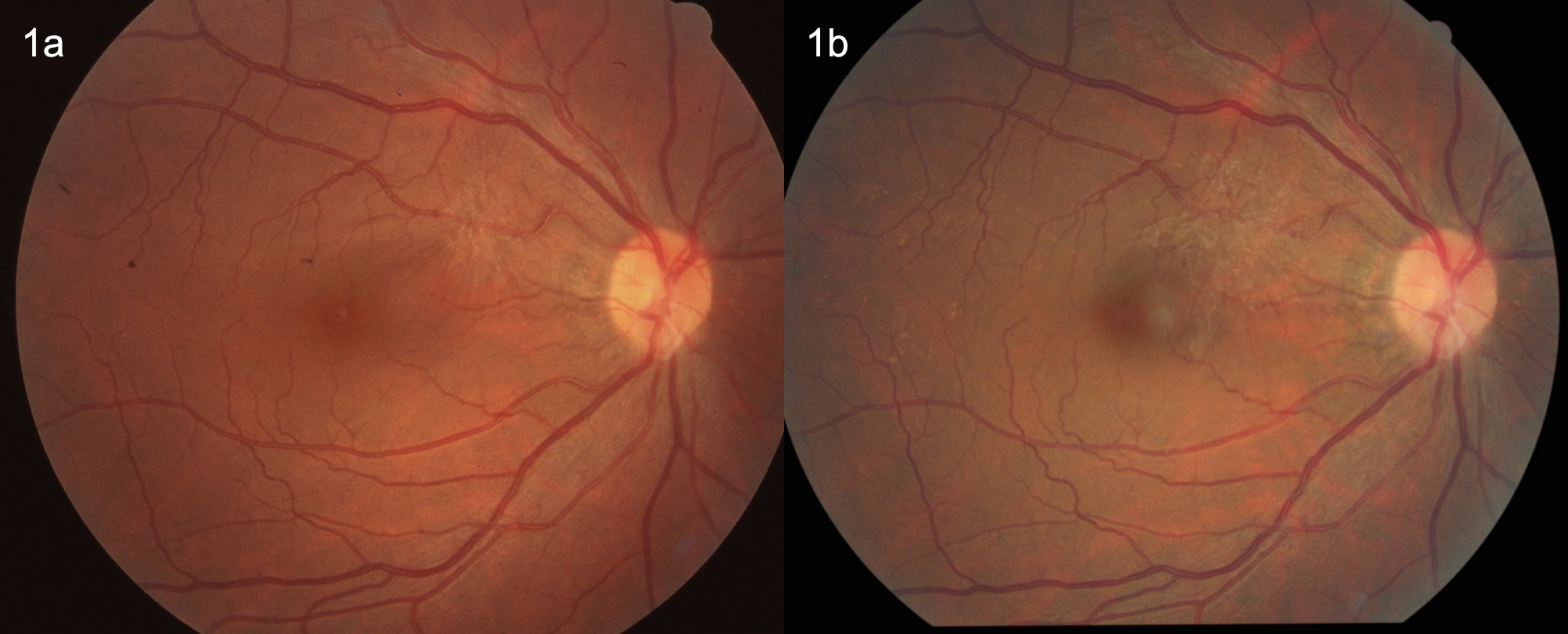
Fig 1A: Right eye of 55-year-old man with early preretinal macular fibrosis superior to the fovea at baseline in 2001. Fig 1B: After 10 years, the area of preretinal macular fibrosis is enlarged.

**Fig 2:**
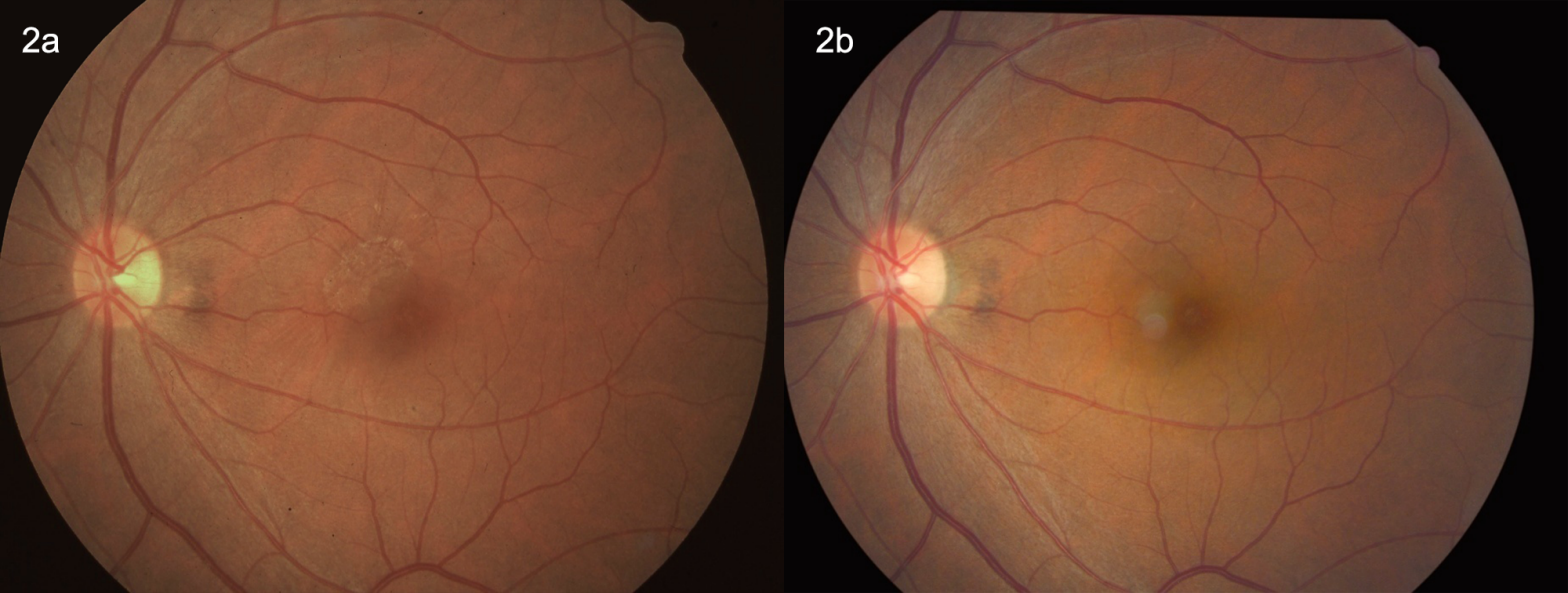
Fig 2A: Left eye of a 47-year-old woman with early preretinal macular fibrosis superior to the fovea macula at baseline in 2001. Fig 2B: After 10 years, the preretinal macular fibrosis disappeared.

**Fig 3:**
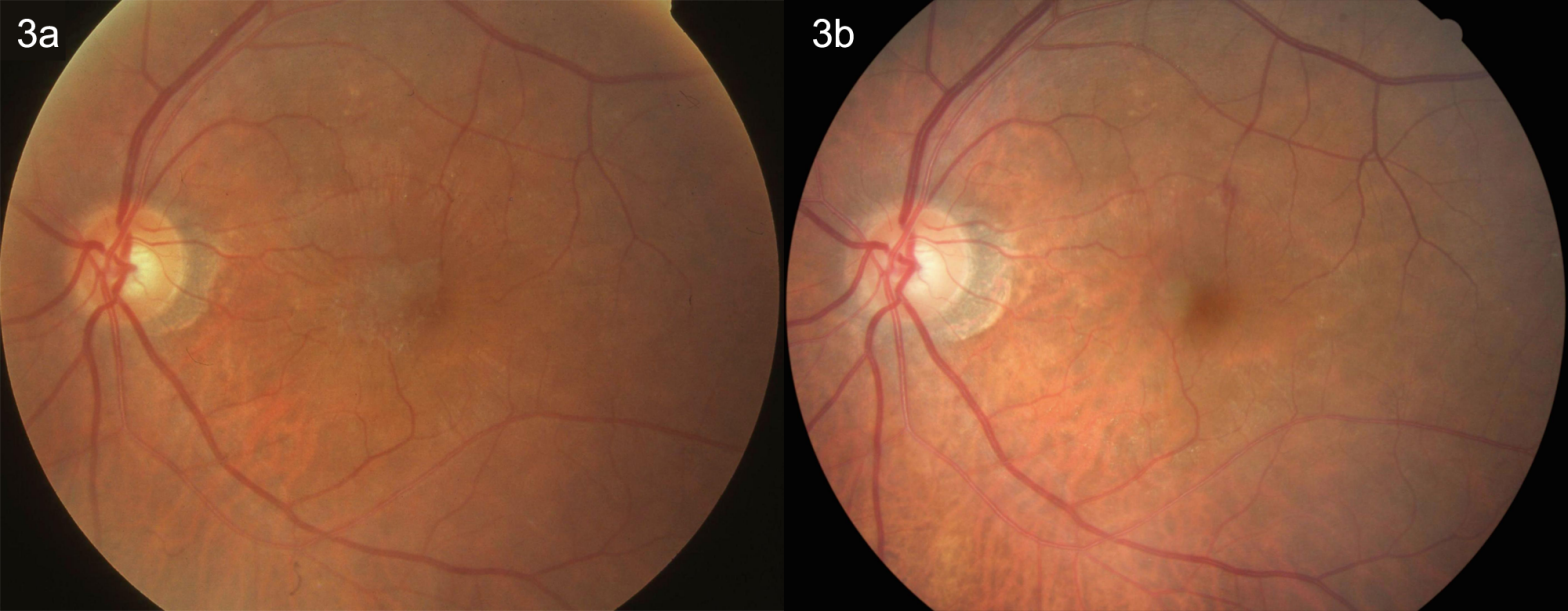
Fig 3A: Left eye of a 76-year-old man with preretinal macular fibrosis at the baseline examination in 2011. Fig 3B: After 10 years, the preretinal macular fibrosis transformed to cellophane macular reflex, with cataract surgery performed in 2006.

**Fig 4:**
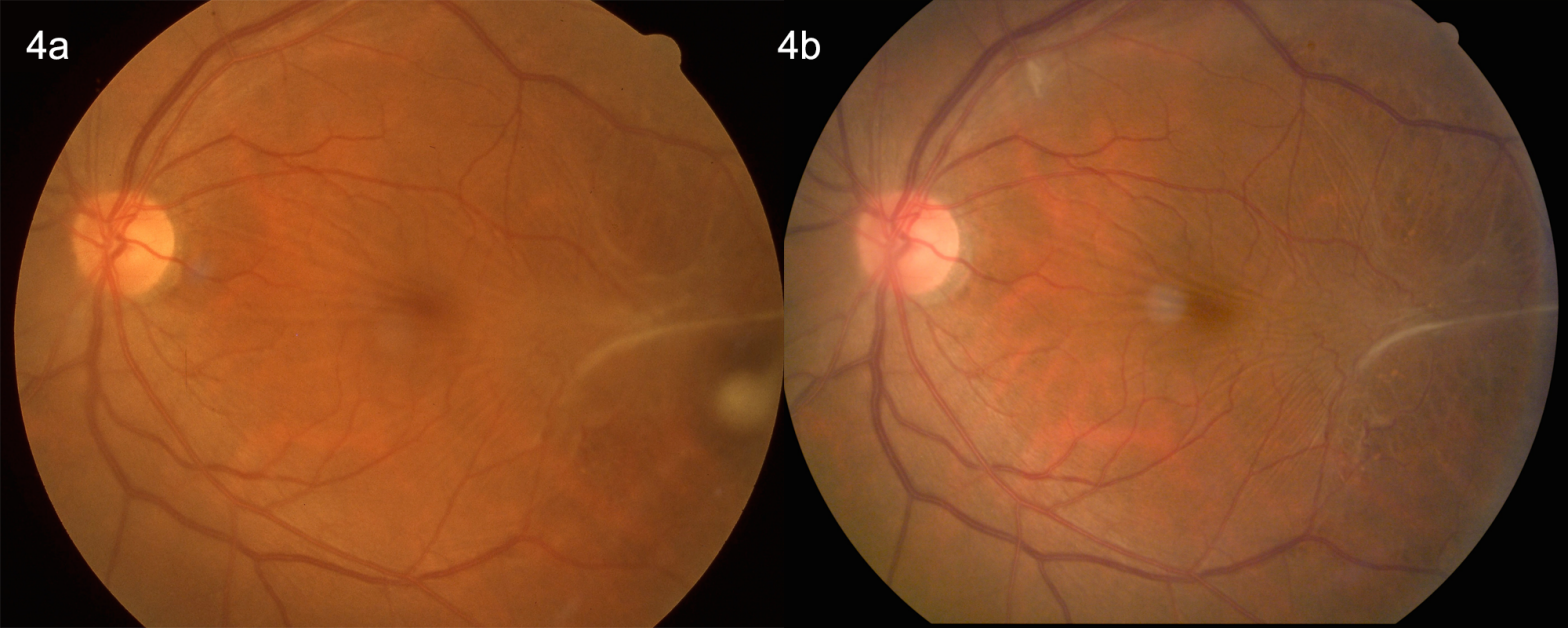
Fig 4A: Left eye of a 69-year-old woman with preretinal macular fibrosis at the baseline examination in 2001. Fig 4B: After 10 years, the epiretinal membrane remained unchanged.

Using the fundus photographs (non-stereoscopic 45°photograph of the central fundus and of the optic disc), ERMs were assessed in a masked manner. The retinal photographs were examined by two ophthalmologists (YY, YNY) with retinal subspecialty training for the presence of ERM. In case of doubt, a panel (YY, YNY, YXW, JBJ) made the final decision. The ERMs were graded using the method described by Klein and colleagues which divided the ERMs into two types, cellophane macular reflex (CMR) and premacular fibrosis (PMF) [1]. The CMR as the early form of ERMs was characterized by a patch or patches of irregular increased reflections from the inner retinal surface and was usually asymptomatic. The PRF as the more severe form of ERMs showed a retinal surface wrinkling and could be associated with significant loss of visual acuity [1,2]. Participants with both CMR and PMF were allocated to the PMF group. Progression of ERM was defined by an enlargement of the area of CMR or PMF by more than 25%, or a conversion of CMR to PMF (Fig 1). Regression of ERM was defined by a decrease of more than 25% in the area involved, by a disappearance of the ERM disappeared or by a regression of the ERM from PMF to CMR (Figs 2 and 3). The ERMs were graded as stable if the change in the area involved was less than 25% (Fig 4). The area of the ERM was assessed by Image J software (https://imagej.net/Downloads; assessed 31.12.2017).

The statistical analysis was performed using a commercially available statistical software package (SPSS for Windows, 22.0, IBM-SPSS, Chicago, IL). In a first step, we determined the mean values (presented as mean ± standard deviation) of the main outcome parameters. In a second step, we performed univariate analyses of the associations between the incidence of ERM and other systemic parameters and ocular parameters. In a third step, we carried out multivariate binary regression analyses with the incidence of ERM as the dependent parameter and with all those variables as independent parameters that were significantly associated with the incidence of ERM in the univariate analyses. Odds ratios (ORs) and 95% confidence intervals (CI) were presented. A *P*-value < 0.05 was considered to indicate statistical significance.

## Results

The baseline examination in 2001 included 4439 participants out of whom 379 individuals died during the study period. Out of the group of the remaining individuals (n = 4060), 1365 subjects were not available for a re-examination in 2011, since they either did not agree to be re-examined or since they had moved away. The present study thus primarily assessed 2695 people or 66.4% of the surviving participants of the baseline examination. These individuals were significantly younger than the non-participants (age (in 2001): 54.5 ± 9.7 years versus 57.0 ± 10.8 years; *P*<0.001) and lived more often in the rural region (48.6% versus 42.3%; *P*<0.001). Both groups did not vary significantly in refractive error (-0.35 ± 2.14 diopters versus –0.34 ± 2.18 diopters; *P* = 0.23) and gender (men: 42.0% versus 44.0%; *P* = 0.06). In a second step, we excluded out of the 2695 subjects all individuals without fundus photographs of sufficient quality in 2001 or in 2011 (129 subjects), so that finally 2566 subjects were included into the study.

For the assessment of the incidence, we included all 2476 individuals (1057 (42.7%) men) with no ERM at baseline. Their mean age was 69.0 ± 7.8 years (median: 69.0 years; range: 51 to 93 years), their mean refractive error was -0.24 ± 1.82 diopters (median: 0.00 diopters; range: -5.5 to +10.00 diopters), and their mean axial length was 23.32 ± 0.93 mm (median: 23.21 mm; range: 19.92–26.33 mm).

At the end of the follow-up, incident ERMs were observed in 208 individuals (335 eyes) out of the 2476 subjects with no preexisting lesions in either eye at baseline. The incidence rate was 8.4% (95% CI: 7.4, 9.5). In 50 persons (24%), the ERMs newly developed in both eyes, and in 158 persons (76%), incident ERMs were detected unilaterally. The incidence of PMFs with 2.5% (95% CI: 1.9, 3.1) was lower than the incidence of CMRs with 5.9% (95% CI: 5.0, 6.9). The incidence of ERMs increased with older age (Table 1). It was was 3.1% among participants younger than 60 years, 10.0% for persons aged 60 to 69 years, 14.4% for persons aged 70 to 79 years, and 10.9% for individuals aged 80+ years (Table 1). The incidence of ERMs peaked in the age group of 70 to 79 years. The incidence of any ERM did not differ significantly between men and women (7.5% versus 9.1%) after adjusting for age (OR: 1.24; 95% CI: 0.92, 1.66).

**Table 1:** Study population and 10-year incidence of epiretinal membranes, stratified by age and gender, in the Beijing Eye Study 2001/2011.

	Age Group (Years)				Gender		All Study Particpants
Type of Epiretinal Membrane	<60	60–69	70–79	>80	Women	Men	
	(n = 972)	(n = 777)	(n = 589)	(n = 138)	(n = 1418)	(n = 1058)	(n = 2476)
Cellophane Macular reflex	21	55	59	12	94	53	147
Preretinal Macular Fibrosis	9	23	26	3	35	26	61
Total	30 (3.1%)	78 (10.0%)	85 (14.4%)	15 (10.9%)	129 (9.1%)	79 (7.5%)	208 (8.4%)

In univariate analysis, a higher incidence of ERM was significantly associated with age (*P*<0.001), shorter body height (*P* = 0.04), lower diastolic blood pressure (*P* = 0.01), rural region of habitation (*P* = 0.007), higher prevalence of aspirin intake (*P* = 0.03), worse best corrected visual acuity (*P*<0.001), lower intraocular pressure (*P* = 0.03), higher prevalence of arcus senilis (*P*<0.001), deeper anterior chamber depth (*P* = 0.025), higher prevalence of previous cataract surgery (*P*<0.001), higehr prevalence of nuclear cataract (*P*<0.002), and higher prevalence of complete posterior vitreous detachment (*P*<0.001) (Table 2).

**Table 2:** Associations (univariate analysis) between the ten-year incidence of epiretinal membranes and ocular and systemic parameters in the Beijing Eye Study 2001/2011.

Parameter	*P*-*Va*lue	Odds Ratio	95% Confidence Interval
General Parameters			
Age (Years)	<0.001	1.06	1.05–1.08
Gender (Men / Women)	0.15	1.24	0.93–1.66
Rural / Urban Region of Habitation	0.007	1.49	1.11–1.98
Education (High school and Higher)	0.62	1.02	0.94–1.10
Cognitive Score	0.65	1.01	0.97–1.06
Body Height (cm)	0.04	0.98	0.96–1.00
Body Weight (kg)	0.09	0.99	0.98–1.00
Waist Circumference (cm)	0.50	1.00	0.99–1.02
Systolic Blood pressure (mmHg)	0.40	1.00	0.99–1.01
Diastolic Blood pressure (mmHg)	0.01	0.99	0.97–1.00
Hyperlipidemia	0.12	0.97	0.93–1.01
Arterial Hypertension	0.11	1.28	0.94–1.73
Diabetes	0.11	1.41	0.93–2.13
History of Cardiovascular Disease	0.43	1.16	0.80–1.70
History of Cerebral Infarction	0.28	1.35	0.79–2.32
Aspirin Intake	0.03	1.40	1.04–1.90
Smoking	0.19	0.80	0.57–1.12
Body Mass Index >25.00 kg/m^2^	0.27	0.85	0.63–1.14
Glycosylated Hemoglobin HbA1c (%)	0.81	0.97	0.79–1.21
Serum Concentration of Glucose (mmol/L)	0.14	0.88	0.75–1.04
Serum Concentration of Creatinine (μmol/L)	0.79	1.00	0.99–1.01
Serum Concentration of Cholesterol (mmol/L)	0.12	1.13	0.97–1.33
Serum Concentration of High-Density Lipoproteins (mmol/L)	0.19	1.27	0.89–1.80
Serum Concentration of Low-Density Lipoproteins (mmol/L)	0.12	1.17	0.96–1.41
Serum Concentration of Triglycerides (mmol/L)	0.70	0.98	0.88–1.09
C-Reactive Protein	0.26	0.95	0.88–1.04
Ocular Parameters			
Best Corrected Visual Acuity (logMAR)	<0.001	0.39	0.24–0.64
Refractive Error (Diopters)	0.46	1.03	0.95–1.11
Intraocular Pressure (mmHg)	0.03	0.94	0.89–0.99
Axial Length (mm)	0.37	1.06	0.93–1.20
Anterior Chamber Depth (mm)	0.03	1.36	1.04–1.78
Cornea Thickness (μm)	0.21	1.00	1.00–1.01
Subfoveal Choroidal Thickness (<250um)	0.05	1.40	1.00–1.80
Cataract Surgery	<0.001	3.94	2.54–6.12
Arcus Senilis	<0.001	1.44	1.22–1.69
Retinal Vein Oclussion	0.07	1.92	0.94–3.94
Retinal Neural Fiber Layer Defects	0.54	1.17	0.71–1.90
Complete Posterior Vitreous Detachment (Detected by Optical Coherence Tomography)	<0.001	2.52	1.69–3.74
Glaucoma	0.27	1.37	0.78, 2.38
Open-Angle Glaucoma	0.20	1.59	0.78, 3.25
Angle-Closure Glaucoma	0.81	0.84	0.20, 3.57
Age-Related Macular Degeneration, Any Stage	0.12	1.28	0.94, 1.74
Age-Related Macular Degeneration, Early Stage	0.18	1.37	0.86, 2.19
Age-Related Macular Degeneration, Intermediate Stage	0.43	1.16	0.80, 1.67
Age-Related Macular Degeneration, Late Stage	0.67	0.67	0.10, 4.33
Diabetic Retinopathy, Prevalence	0.46	1.39	0.59, 3.28
Diabetic Retinopathy, Stage	0.56	1.13	0.76, 1.67
Cataract, Nuclear	0.002	1.70	1.21, 2.38
Cataract, Cortical	0.66	1.11	0.71, 1.73
Cataracta, Subcapsular Posterior	0.22	0.48	0.15, 1.55

The binary regression analysis included the incidence of ERM as dependent variable and as independent variables all those parameters which were significantly associated with the ERM incidence in the univariate analysis. We then dropped parameters which were no longer significantly associated with the incidence of ERMs, starting with the parameters with the highest *P*-values. In the final model, a higher incidence of ERMs remained to be significantly associated with older age (*P*<0.001), previous cataract surgery (*P* = 0.003) and presence of a complete posterior vitreous detachment (*P* = 0.02) (Table 3).

**Table 3:** Associations (multivariate analysis) of the ten-year incidence of epiretinal membranes in the Beijing Eye Study 2001/2011.

Parameter	*P*- Value	Odds Ratio	95% Confidence Interval
Age (Years)	<0.001	1.06	1.04–1.09
Age Group (60–69 years old)	<0.001	2.87	1.76–4.67
Age Group (70–79 years old)	<0.001	4.02	2.25–7.18
Cataract Surgery	0.003	3.32	1.51–7.29
Complete Posterior Vitreous Detachment	0.02	1.84	1.12–3.02

For 48 eyes out of 112 eyes (90 study participants) with pre-existing ERMs at baseline in 2001, the fundus photographs taken in 2011 could not sufficiently be evaluated. Out of the remaining 64 eyes, 20 (31.3%) eyes showed a progression, 20 (31.3%) eyes were stable, and 24 (37.5%) showed a regression of the ERMs (Table 4). Among the 24 eyes with ERM regression, the ERM area decreased by more than 25% or the ERM type changed from PMF to CMR in 15 eyes (23.4%) or the ERM completely disappeared in 9 eyes (14.1%). The rate of progresison or regression of pre-existing ERMs was not signifcantly associated with any other ocular or general parameter.

**Table 4:** Change in epiretinal membranes in eyes with pre-existing epiretinal membranes during a 10-year follow-up in the Beijing Eye Study 2001/2011.

Type of Epiretinal Membrane at Baseline			
Change during 10-Year Period	Preretinal Macular Fibrosis (n = 36 eyes)	Cellophane Macular Reflex (n = 28 eyes)	Any (n = 64 eyes)
Progressed	14 (38.9%)	6 (21.4%)	20 (31.3%)
Stable	10 (27.8%)	10 (35.7%)	20 (31.3%)
Regressed	12 (33.3%)	12 (42.9%)	24 (37.5%)

## Discussion

In this population-based longitudinal study on a Chinese population aged 40+ years, the frequency of newly developed ERMs during a period of 10 years was 208 / 2476 or 8.4% (95% CI: 7.4, 9.5). The ERMs newly developed In 50 persons (24%), in both eyes and in 158 persons (76%) unilaterally. The incidence of PMFs (2.5%; 95% CI: 1.9, 3.1) was lower than the incidence of CMRs (5.9%; 95% CI: 5.0, 6.9). The incidence of ERMs increased with older age with a peak in the age group of 70 to 79 years, previous cataract surgery and complete posterior vitreous detachment.

The 10-year incidence of ERMs of 8.4% as detected in our study was higher than the 5-year incidence of ERMs of 5.3% as examined in the Blue Mountains Eye Study [3]. Parallel to previous cross-sectional studies on the prevalence of ERMs, we did not observe a statistically significant sex difference in the incidence of ERMs [12,13,15]. In contrast, the Singapore Malay Eye Study reported on a higher prevalence of RMs in women [10]. Our observation of an association between a higher incidence of ERMs and older age, previous cataract surgery and complete posterior vitreous detachment was in agreement with the results of studies on the prevalence of ERMs which showed similar relationships [1,7,8,11–13,17]. The relative decrease in the incidence of ERMs for the oldest age group as compared to the age group of 70 to 79 years in our study population might have been caused by increasing, yet unoperated cataract, preventing a clear view onto the retinal surface. Correspondingly, the Singapore Epidemiology of Eye Disease Study revealed that approximately 80% of pseudophakic elderly particpants showed an ERM [23]. Similarly, a study by Fong and colleagues showed that in 1040 study participants undergoing cataract surgery and having retinal photographs taken preoperatively and at one month postoperatively, ERMs were detected in 3.1% preoperatively and in 14.8% postoperatively [24]. It suggested that a substantial under-detection of ERMs migh thave occurred due to the presence of cataract. Posterior vitreous detachment has been considered to play a key role in the pathogenesis of ERMs [25]. It fits with the observation made in our study population on a relationship between a higher incidence of ERMs and a higher prevalence of a posterior vitreous detachment. In that context, one may also take into account that cataracat surgery can induce posterior vitreous detachment, indirectly leading to the development of an ERM.

In our study, we did not detect significant associations between ocular and systemic parameters and the progression or regression of ERMs. This lack of associations might have been due to the relatively low number of participants showing a progression or regression of ERMs, so that the statistical power for this analysis might have been too low.

Limitations of our study should be discussed. First, detection of ERMs is markedly easier and more precise if performed by optical coherence tomography (OCT) than by the assessment of fundus photographs. Since however the OCT technology was not yet available in 2001, a ten-year follow study had to rely on fundus photographies. Future prospective longitudinal studies may apply the OCT methodology which may allow to simultaneously assess the presence of a partial or complete posterior vitreous detachment and to explore relationships between longitudinal changes in the vitreo-retinal interface and the development or change in ERMs. Second, as for any population-based study, non-participation might have led to a bias. The rate of participation in our study was however relatively high, with originally 83.4% of the eligible individuals participating in the baseline examination and with 66.4% of the surviving participants of the baseline examination returning for the 10-year follow-examination. Third, since the Beijing Eye Study included Chinese, the results may not directly be transferred on other ethnicities.

In conclusion, in this population-based longitudinal study on a Chinese population aged 40+ years, the incidence of newly developed ERMs during a period of 10 years was 8.4% (95% CI: 7.4, 9.5). The majority of the new ERMs developed unilaterally (158 (76%) individuals). The incidence of PMFs (2.5%) was lower than the incidence of CMRs (5.9%). The incidence of ERMs increased with older age with a peak in the age group of 70 to 79 years, with a higher prevalence of previous cataract surgery and with a higher prevalence of a complete posterior vitreous detachment.
